# Removal of Heavy Metal Ions from Wastewater with Poly-ε-Caprolactone-Reinforced Chitosan Composite

**DOI:** 10.3390/polym14235196

**Published:** 2022-11-29

**Authors:** Manuel E. Martínez, José René Rangel-Méndez, Miquel Gimeno, Alberto Tecante, Gretchen T. Lapidus, Keiko Shirai

**Affiliations:** 1 Laboratorio de Biopolímeros y Planta Piloto de Bioprocesos de Residuos Agroindustriales y de Alimentos, Unidad Iztapalapa, Departamento de Biotecnología, Universidad Autónoma Metropolitana, Av. Ferrocarril San Rafael Atlixco número 186, Colonia Leyes de Reforma 1a sección, Alcaldía de Iztapalapa, Mexico City 09310, Mexico; 2 División de Ciencias Ambientales, Instituto Potosino de Investigación Científica y Tecnológica, A.C., Camino a la Presa San José No. 2055, San Luis Potosi 76210, Mexico; 3 Departamento de Alimentos y Biotecnología, Facultad de Química, Universidad Nacional Autónoma de México, Cd. Universitaria, Mexico City 04510, Mexico; 4 Unidad Iztapalapa, Departamento de Ingeniería de Procesos e Hidráulica, Universidad Autónoma Metropolitana, Avenida Ferrocarril San Rafael Atlixco número 186, Colonia Leyes de Reforma 1a Sección, Alcaldía de Iztapalapa, Mexico City 09310, Mexico

**Keywords:** chitosan, poly-ε-caprolactone, composite, metal ions removal, adsorption

## Abstract

Currently, the requirements for adsorbent materials are based on their environmentally friendly production and biodegradability. However, they are also related to the design of materials to sustain many cycles in pursuit of low cost and profitable devices for water treatments. In this regard, a chitosan reinforced with poly-ε-caprolactone thermoplastic composite was prepared and characterized by scanning electron microscopy; Fourier transforms infrared spectroscopy, X-ray diffraction analysis, mechanical properties, as well as erosion and swelling assays. The isotherm and kinetic data were fitted with Freundlich and pseudo-second-order models, respectively. The adsorption equilibrium capacities at pH 6 of Zn(II), Cu(II), Fe(II), and Al(III) were 165.59 ± 3.41 mg/g, 3.91 ± 0.02 mg/g, 10.72 ± 0.11 mg/g, and 1.99 ± 0.22 mg/g, respectively. The adsorbent material lost approximately 6% of the initial mass in the adsorption-desorption processes.

## 1. Introduction

Metals or metalloids such as Pb, Cd, Hg, As, Cr, Cu, Se, Ni, Ag, and Zn, as well as other metallic contaminants such as Al, Cs, Co, Mn, Mo, Sr, and U are very stable and difficult to remove by chemical or biological means. The concentration of heavy metals in the environment varies depending on the type of anthropomorphic activity such as mining, electroplating, and smelting industrial processes [1], alongside the management of these wastes to avoid leaching of the metal ions from the soil into surface and groundwater bodies [2].

One of the most commonly present ions in aqueous media is Zn which is released in the environment through the effluents of industrial activities such as galvanization, pigment formation, and the production of stabilizers, thermoplastics, alloys, and batteries [3,4,5]. Although living organisms, humans and animals, require Zn, excessive levels result in health issues and environmental damage [6,7]. In this regard, the World Health Organization (WHO) has recommended a maximum Zn concentration for drinking water of 3 mg L^−1^ [3,8]. According to the US EPA, the maximum limit concentration of water to preserve aquatic freshwater organisms is 120 μg L^−1^, while for human health it is 7400 μg L^−1^ [9,10]. The European Union has registered Zn in the Dangerous Substances Directive as a List 2 set at 40 μg L^−1^ for estuarine and marine waters and 500 μg L^−1^ as an environmental quality standards [5,11]. Therefore, several methods are investigated and applied to remove metal ions from contaminated water, such as bioleaching, biodegradation, chemical precipitation, ion exchange, membrane filtration, electrochemical, and adsorption [12]. The common problem with combined methods such as oxidation reactions is that they can be relatively slow and ineffective when metal concentrations are low. In contrast, electrochemical and membrane filtration methods have good removal yields but require significant investments and energy consumption. Coagulation and flocculation are less expensive and simpler procedures than the others, but they involve the consumption of chemicals with the production of a large amount of difficult-to-treat sludge. The adsorption process for heavy metal removal employ activated carbons, polymeric organic resins, activated alumina, silica gel, zeolites, and molecular sieves that are used as broad-spectrum adsorbents, although expensive and rapidly saturated with limited regeneration [13,14]. Their null or low biodegradability is also a drawback since they become waste after their useful life. There are also non-conventional, cheap, and readily available adsorbents from agricultural, forest, and fishery biomasses [12]. Chitosan (CS), a copolymer of glucosamine and *N*-acetyl-glucosamine, obtained by chitin deacetylation, is used in water treatment as a coagulant and adsorbent [15]. The protonation of the amine groups of CS at an acidic pH promotes interactions with negatively charged contaminants from aqueous effluents by anion complexation and coagulation-flocculation processes. At a neutral pH, CS coordinates to metal ions either by the chelation process, electrostatic attraction, or by forming ternary complexes with other organic pollutants present in wastewater effluents [16]. Limitations exist, nonetheless, due to the acidic pH in most wastewater effluents at which protonated forms affect its selectivity in addition to CS mass losses in the solid-liquid adsorption process [17]. To overcome these constraints, chemical modifications, such as grafting [4], cross-linking [18], surfactant modifications [14], and mixing CS with filtering materials such as diatomaceous earth [19] and other CS-based composites [20], have been investigated to improve heavy metal removal. Aqueous Zn batteries are another example of the adsorption of hydroxyl, amino, and ether CS groups with protons, zinc ions, and sulfates in ZnSO_4_ electrolytes that avoid undesirable side reactions and induce uniform Zn deposition [21]. Despite the benefits of CS, the combination with biodegradable polyester to counteract its poor mechanical properties has been reported elsewhere [20]. In this regard, poly-ε-caprolactone (PCL) is used alone or in combination with other polymers to improve the flexibility and processability [22]. A composite design and its characterization are aimed in this study using the cost-effective manufacturing process of thermoplastic extrusion. The criteria to select the formulation were the well-known CS adsorption capacity and PCL cost, the latter is more expensive than the former, as well as the enhancement of mechanical properties. As a result, metal ions can be removed from water by the adsorbent thermoplastic matrix composite of CS:PCL (MCP) with minimal mass loss during adsorption-desorption, and thus it is reusable, made with biodegradable polymers, and environmentally friendly.

## 2. Materials and Methods

### 2.1. Materials

CS was obtained by thermochemical deacetylation of raw chitin extracted by lactic acid fermentation (*Lactobacillus brevis*) of shrimp wastes (*Litopenaeus vanameii*) as previously reported [23]. The degree of acetylation for CS was 7.94% with a viscosimetric molecular mass of 232 kDa. In addition, PCL was enzymatically synthesized following the procedure reported by García-Arrazola et al. [24] as a white coarse powder with a number-average molecular mass (Mn) of 30 kDa as determined by GPC/SEC vs. PS standards.

### 2.2. Ch-PCL Thermoplastic Matrix Composite (MCP)

MCP was prepared by mixing CS with PCL in a mass ratio of 2:1, 1:1 and 1:5 in a temperature-controlled bath of 70 °C until homogenization of materials was reached. The extrusion was carried out in a LE-075 lab-scale extruder (Custom Scientific Instruments, Easton, PA, USA) with the rotor temperature at 65 °C and head at 75 °C [20] to obtain cylindrical pellets with a 1.97 ± 0.07 mm mean diameter and 6.4 ± 1.2 mm height. According to adsorption efficiencies (Appendix A), only the formulation 2:1 was used henceforth for characterization, adsorption and desorption process.

### 2.3. Polymers and MCP Characterization

The chemical structures of the materials were analyzed by ATR-FTIR in a Spectrum 100 (Perkin Elmer, Boston, MA, USA). The percentage of crystallinity (*C%*) was determined by powder X-ray diffraction (XRD) in a D8 Advance Davinci (Bruker, Billerica, MA, USA). The pH-zpc of MCP was determined by potentiometric titrations in a T50 (Mettler Toledo, Columbus, OH, USA). The tensile strength, maximum elongation at break, and Young’s modulus were measured in a MTS Sintech 1/S (MTS System Corporation, Prairie, MN, USA). Elemental analysis was performed using energy-dispersive X-ray spectroscopy (EDS) (Oxford Instrument, Abingdon, UK) [20].

Microscopic analyses were carried out by treating a thin layer of the cross-section of MCP (0.5 mm) suspended in 3 mL of phosphate buffer (pH 7.2) with 30 μL of calcofluor white-reagent (CW) and vortexing for 20 s. After 2 h of reaction, samples were separated by decantation and the thin layer was observed under a microscope with fluorescent light in a microscope Axiostar plus (Carl Zeiss, Jena, Germany). Scanning electron micrographs (SEM) for MCP, CS, and PCL were obtained from a microscope JEOL JSM-5900 lv (Tokyo, Japan).

The average pore size was determined from the cross-sections of the MCP samples (six samples) and converted to 8-bit black and white images, in which the white areas represent the pores and black areas represent the solid surface. Binary images were analyzed as particles employing ImageJ 1.53a (National Institutes of Health, Bethesda, MD, USA) to determine surface porosity and average pore size (APS) [25]. The adsorption-desorption isotherm of N_2_ to 77 K technique was also used to determine the MCP’s specific surface area and pore volume in a Micro 200 (3P Instruments, Leipzig Germany). MCP degasification was performed for 12 h at 313.15 K. The specific surface area was calculated using Equation (1) [26], from the monolayer capacity, $V_{m}^{a}$, expressed in cm^3^ (STP)/g units,

(1) $$a_{s}=\frac{V_{m}^{a}L\sigma_{m}}{2.24\times 10^{22}}$$

where *L* is the Avogadro’s number and *σ_m_* is the molecular area occupied by an adsorbed N_2_ molecule in the complete monolayer that commonly has a value of 0.162 nm^2^. The monolayer capacity, $V_{m}^{a}$, and the energetical parameter, *C*, were determined by linearization of the Brunauer–Emmett–Teller (BET) Equation (2) [26]:

(2) $$\frac{p/p^{0}}{V\left(1-p/p^{0}\right)}=\frac{1}{V_{m}C}+\frac{C-1}{V_{m}C}\left(\frac{p}{p^{0}}\right)$$

where *V* is the specific amount of N_2_ adsorbed at the relative pressure (*p*/*p*^0^) per gram of adsorbent.

The erosion (*E%*) of MCP (29 ± 2 mg) was determined by placing 10 mL of deionized water in a flask and adding 1 N HCl until reaching a pH of 4. Composites were filtered, dried, and weighed every 24 h to determine the mass loss using Equation (3):

(3) $$E\%=\frac{X_{0}-X_{E}}{X_{0}}\times 100$$

where *X*_0_ is the mass of the dry pellet before contact with acidic water, and *X_E_* is the mass after being in contact with acidic water. Similarly, the swelling capacity of MCP (*S%*) was determined by placing the sample (29 ± 2 mg) in screw-top tubes, adding 10 mL of deionized water, and placing them in a controlled bioclimatic chamber HPP260 (Memmert, Schwabach, Germany) at 25 °C with a relative humidity (RH) of 45%. MCP was collected every 24 h, placed on Whatman filter paper to remove the superficial water, and weighted (*X_S_*). All measurements were conducted in triplicate. Equation (4) was used to calculate the amount of water retained in the pellets:

(4) $$S\%=\frac{X_{S}-X_{0}}{X_{0}}\times 100$$

### 2.4. Adsorption Experiments

#### 2.4.1. Adsorption Equilibrium Studies

Solutions with concentrations of Cu(II) (20–120 mg L^−1^), Fe(II) (15–90 mg L^−1^), and Zn(II) (70–420 mg L^−1^) were prepared at a pH of 6. Cu(NO_3_)_2_, Pb(NO_3_)_2_, ZnSO_4_, FeSO_4_, Cd(NO_3_)_2_, and Al(NO_3_)_3_ were dissolved in distilled water and adjusted to a pH of 6 with NaOH (1.25 N). The calibration curves for Cu(II), Pb(II), Zn(II), Fe(II), Cd(II), and Al(III) were determined by atomic absorption spectroscopy (AAS) in a Varian 200FS (USA). Experiments were carried out in 15 mL glass flasks by mixing 0.01 g of MCP with 10 mL of metal ion solution in an orbital shaker (50 rpm) at 25 °C [27]. Experiments were halted after 120 min to ensure that equilibrium was reached. AAS determined the heavy metal concentrations in the samples, and the adsorption capacity (*q_t_*) (mg g^−1^ MCP) was estimated by the Equation (5):

(5) $$q_{t}=\frac{\left(C_{0}-C\right)v}{m}$$

where *C*_0_ is the initial concentration of each heavy metal, *C* is the remaining heavy metal concentration in the solution after each time, *v* is the volume of sample, and *m* is the mass of MCP used in each experiment.

The linearized Freundlich model (Equation (6)), the Langmuir model (Equation (7)), and the Dubinin-Radushkevich model (Equation (8)) were employed to fit the adsorption isotherms [28]:

(6) $$\log q_{eq}=\log k_{F}+\log C_{eq}\left(\frac{1}{n}\right)$$

where *q_eq_* is the equilibrium adsorption capacity (mg g^−1^), *k_F_* is the adsorption affinity constant (L mg^−1^), *n* represents the system’s heterogeneity and *C_eq_* is the concentration of the metal ions (mg L^−1^) at equilibrium.

(7) $$\frac{C_{eq}}{q_{eq}}=\frac{C_{eq}}{q_{max}}+\frac{1}{k_{L}q_{max}}$$

where *q_max_* is the maximum adsorption capacity (mg g^−1^) and *k_L_* is the Langmuir constant (L mg^−1^).

(8) $$\log q_{eq}=\log q_{max}+k_{DR}\epsilon^{2}$$

where *k_DR_* is the Dubinin-Radushkevich constant (mol^2^ kJ^−2^), and ϵ is the adsorption potential and is calculated by the Equation (9):

(9) $$\epsilon =RT\ln\left(\frac{C_{s}}{C_{eq}}\right)$$

where *C_s_* is the solubility of the adsorbates (mg L^−1^), *R* is the gas constant (8.31 J mol^−1^ K^−1^) and T is the absolute temperature (298.15 K).

#### 2.4.2. Adsorption Kinetic Studies

Adsorption kinetics were carried out using aqueous synthetic solutions containing heavy metal concentrations above the permissible limits according to the Mexican Official Norm-001 [29] for wastewater discharges into receptive bodies for Cu(II), Pb(II), Zn(II), Fe(II), Cd(II), and Al(III) at 10 mg kg^−1^, 5 mg kg^−1^, 334 mg kg^−1^, 22 mg kg^−1^, 1 mg kg^−1^, and 4 mg kg^−1^, respectively. A total of 0.01 g of MCP was mixed with 10 mL of synthetic solution in an orbital shaker (50 rpm) at 25 °C. Samples for metal ions determination were taken every 5 min for 20 min and thereafter, every 20 min until 100 min. The *q_t_* (mg g^−1^) values were calculated using the Equation (5).

Adsorption kinetic parameters were estimated by fitting the experimental data of *q_t_* to the pseudo-second order (PSO) model (Equation (10)), the Elovich model (Equation (11)) and the pseudo-first order (PFO) model (Equation (12)) [30]:

(10) $$\frac{t}{q_{t}}=\frac{1}{k_{2}\;q_{eq}^{2}}+\frac{t}{q_{eq}}$$

where *q_eq_* and *q_t_* are the adsorption capacities at equilibrium and at time *t* (mg g^−1^), respectively, and *k_2_* (g mg^−1^ min^−1^) is the adsorption rate constant.

(11) $$q_{t}=\left(\frac{1}{\beta }\right)\ln\alpha \beta +\left(\frac{1}{\beta }\right)\ln t$$

where *α* and *β* are the initial sorption rate (mg g^−1^ min^−1^) and the desorption coefficient which is related to the extent of surface coverage and the activation energy for chemisorption (g mg^−1^), respectively.

(12) $$\frac{1}{q_{t}}=\frac{k_{1}}{q_{eq}t}+\frac{1}{q_{eq}}$$

where *k_1_* is the pseudo-first order rate constant (min^−1^).

### 2.5. Desorption and Reusability

Acids or EDTA solutions were used to carry out heavy metal desorption. The acidic condition assays employed HNO_3_ or HCl at concentrations of 0.05, 0.1, or 0.5 N [31,32,33,34]. EDTA solutions (15 g L^−1^) with NaOH 0.25 N (pH 12.8) or 0.5 N (pH 13.6) were also tested [27,35]. MCP samples from the adsorption (Section 2.4.2) were rinsed with deionized water and dried at 30 °C for 24 h. MCP (0.01 g) and 10 mL of desorption solutions were placed in 15 mL glass flasks and mixed in an orbital shaker at 50 rpm for 120 min at 25 °C. The heavy metal concentration in the desorption solutions was determined every 20 min up to 120 min by AAS, and the desorption efficiencies (*ε_des_*) were calculated using Equation (13):

(13) $$\varepsilon_{des}=\left(\frac{C_{des}}{C_{ads}}\right)\times 100$$

where *C_ads_* is the concentration of heavy metals retained in the MCP at equilibrium (100 min) and *C_des_* is the heavy metals concentration in the desorption solutions.

Six cycles were carried out for the reusability of MCP at the conditions mentioned above using for adsorption aqueous synthetic solutions (Section 2.4.2) and, for desorption, the solution with the highest *ε_des_*. The adsorption efficiency (*ε_ads_*) at every cycle was calculated using Equation (14):

(14) $$\varepsilon_{ads}=\left(\frac{C_{ads}}{C_{0}}\right)\times 100$$

## 3. Results and Discussion

### 3.1. Composite Characterization

**Figure 1:**
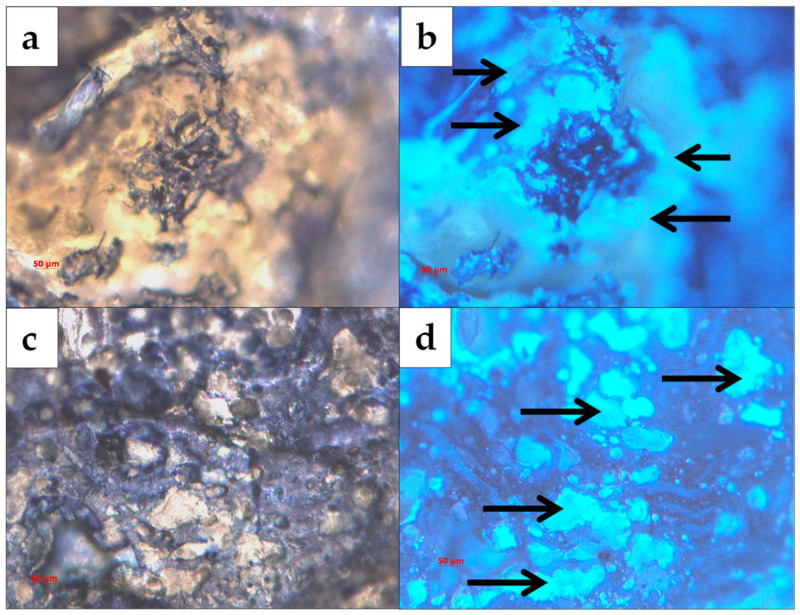
CS-reinforced with PCL (**a**,**c**) and stained with CW (**b**,**d**). Arrows show Ch.

CW staining on the surface and the cross-sections ascertained the distribution of the polymers in the extruded material. Therein, CW bound to the β-1,4 glycosidic bonds of CS and fluoresced, and Figure 1 shows the sheath cluster domains on PCL.

**Figure 2:**
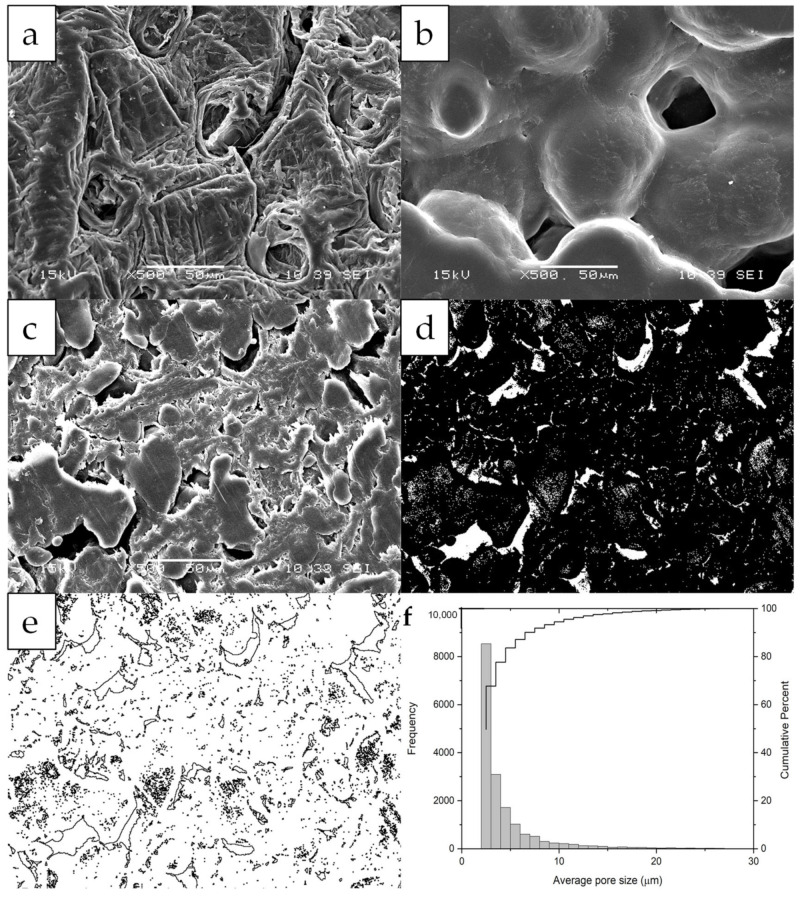
SEM micrographs for CS (**a**), PCL (**b**), MCP (**c**), binary image of MCP (**d**), analysis of particles by ImageJ (**e**) and average pore size distribution (**f**).

The SEM micrographs for CS, PCL, and MCP (Figure 2a–c) evidence that CS and PCL blended heterogeneously in the MCP. The extrusion process drove the melted PCL to settle between solid-milled CS particles (Figure 2c). Similar behavior has been observed by Correlo et al. [36] when CS melted with polyesters showed agglomeration of CS fibers, probably due to insufficient torque during blending. In our work also, during the injection molding, several internal, tiny, and densely distributed pores were formed by aggregation and agglomeration. This influences the bulk density and mechanical properties without a significant contribution to the fluid flow [37]. The low N_2_ adsorbed volume observed in the physisorption isotherms is additional evidence (Appendix B), and hysteresis and pore filling and emptying were not evident. The pore volume of vapor adsorbed, Vp, was 0.004 cm^3^ g^−1^ and the specific surface area resulted in 2.09 m^2^ g^−1^ at a relative pressure close to one (e.g., *p*/*p*0 = 0.98). The MCP displayed open pores having slit shapes with curved channels with one and two ends, which were larger than other closed ones. An APS of 4.68 ± 3.27 μm and a surface porosity of 5.10 ± 1.12% were estimated by image analysis of cross-section SEM micrographs (Figure 2c–f).

**Figure 3:**
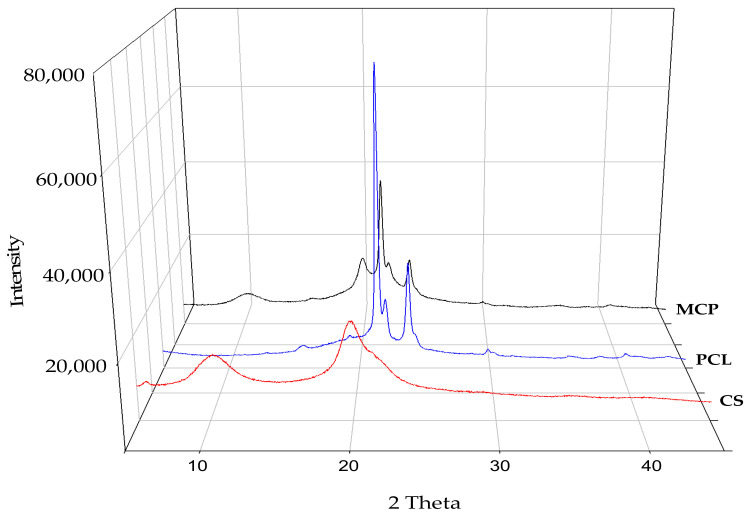
XRD patterns of PCL, CS and MCP.

The XRD pattern for CS showed two characteristic peaks at 11 and 20° assigned to amorphous and crystalline contributions, respectively. The *C%* was 60.83% for this polysaccharide. The crystalline regions of PCL displayed a characteristic sharp peak at 21° with a shoulder at 22° and a lower intensity peak at 23° (Figure 3). The *C%* of PCL was 38.7. Accordingly, an intense band at 1293 cm^−1^ in the FTIR corresponded to C-O-C vibrations for the crystalline PCL phase [38] (Appendix C). The XRD pattern for MCP (Figure 3) revealed that the crystalline and amorphous domains were still present in both materials after the extrusion process. In this regard, Correlo et al. [36] reported that blends of CS and polyesters prepared by injection molding showed weak molecular interactions between the polymers with no influence on the crystalline domains. Pawar and Srivastava [39] also reported that the intensity of the characteristic peaks of PCL in the CS/PCL sponge did not change since the crystal structure of the polymers remained. Of note is that during the deacetylation process, the amine groups increased with a consequent decrease in crystallinity, which might imply an increase in the adsorption capacity, but it could also lead to mass loss due to CS solubility under acidic conditions [40]. In our work, the degree of acetylation of the CS was 7.24% representing a desirable high percentage of amine groups. However, the biological process for the production of CS [23] preserved the crystalline regions (*C%* = 60.83), and therefore, it might prevent excessive mass losses in the MCP at an acidic pH.

**Figure 4:**
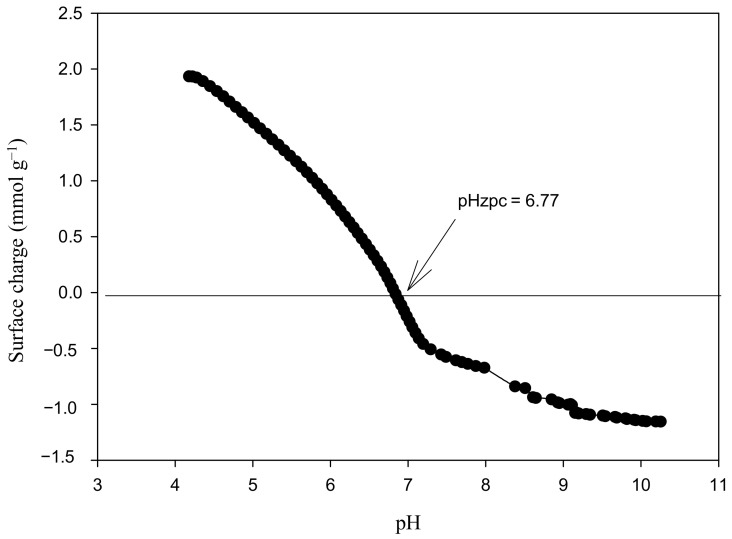
Proton binding curve of MCP.

The proton binding curve of MCP (Figure 4) shows that the pHzpc was 6.7, which is close to the pHzpc of CS (pH 6.3). The surface became negatively charged at pH > pHzpc, thus increasing the metal cations removal efficiency [16]. ZnSO_4_, FeSO_4_, Cd(NO_3_)_2_, and Pb(NO_3_)_2_ dissolved in water provided positively charged metal ions, and the amine groups of MCP were still protonated at a pH of 6, exerting repulsions to a certain extent. Therefore, the electrostatic interactions between the adsorbent and adsorbates might not be relevant in the adsorption process and aid the complex formation of the divalent metal ions with amine, carboxyl, and hydroxyl groups with non-shared electron pairs. It is worth noting that according to Cu speciation at a pH of 6, only a minor fraction remained as a divalent ion, while the major was either in the neutral or solid form, i.e., a small amount adsorbed, and the rest precipitated as salt. Similarly, for Al(NO_3_)_3_, at this pH, the metal speciation was in the passivation state (Appendix D) [41].

**Figure 5:**
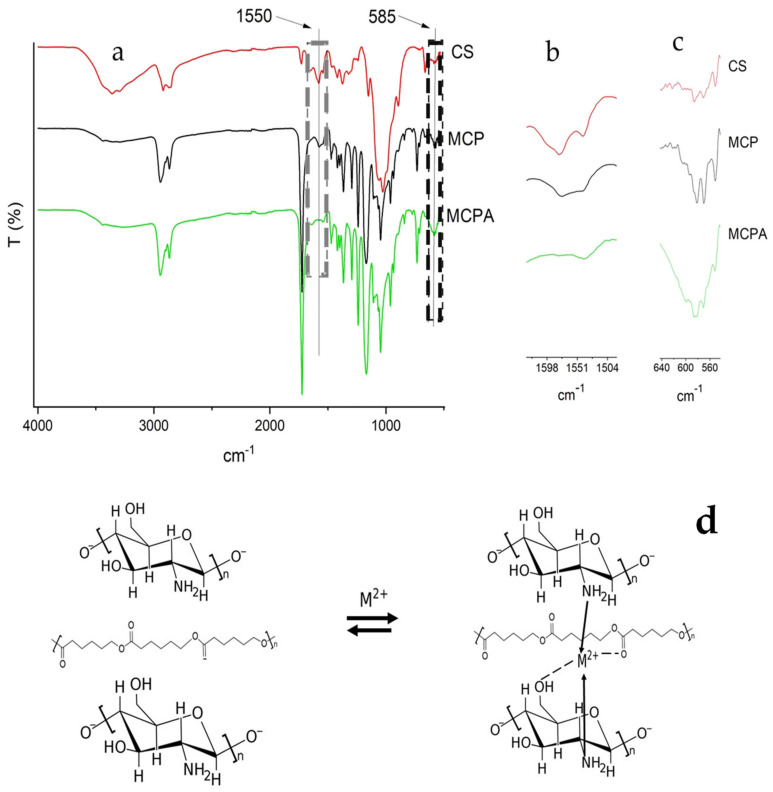
FTIR spectra of CS, PCL, and MCP, before and after adsorption treatment (MCPA) (**a**), 1650–1400 cm^−1^ (**b**), and 650–550 cm^−1^ regions (**c**), and proposed adsorption mechanism (**d**). The grey box represents N-H bending vibrations. The black box shows the stretching vibrations of the metal ion-MCP complex.

The FTIR spectrum for CS displays the characteristic bands at 3260 cm^−1^ assigned to the stretching of the amine, which correlates to the stretching movement of the carbonyl group in the amide chitin bond, 1578 cm^−1^, that concurs with the vibration flexion of methyl in the acetamide group (Figure 5). The characteristic stretching vibrators of PCL were asymmetric and symmetric CH_2_, carbonyl, and C-O-C in the crystalline phase at 2944 cm^−1^, 2865 cm^−1^, 1720 cm^−1^, and 1293 cm^−1^, respectively (Appendix C) [38]. The bands assigned to the amide and amine groups of CS did not decrease in intensity in the FTIR spectrum for MCP. This concludes that these groups did not interact with PCL during the extrusion process and remain available for the adsorption of metal ions.

The FTIR spectra for MCP before and after the adsorption process (MCPA) are shown in Figure 5, which evidence the interactions among the adsorbent and adsorbates. The increase in the intensity band at 580 cm^−1^ is related to Cu-N and Cu-O vibrations [42]. Additionally, the bending vibrations of the amino group at 1559 cm^−1^ decreased and displaced ~9 cm^−1^ after the adsorption process, indicating its involvement in the metal complex formation. The secondary OH of C3 (1076 cm^−1^) and the primary OH of C6 (1027 cm^−1^) related to Cu-O in MCPA were overlapped by bands assigned to PCL. Of note is that the two main mechanisms, bridge and pendant, described for CS-Cu complexation involve two and one nitrogen ligands, respectively [31,43]. Rhazi et al. [44] demonstrated that the formation was more for the pendant than the bridge model, meaning a lower dissociation and greater stability. For the CS-Fe complex, a similar mechanism was described by Bhatia & Ravi [45], where two nitrogen ligands and two oxygen ligands from different CS units were involved in complexation. They also proposed that two more oxygen ligands in the media could stabilize this complex. The appearance of a slight shoulder peak at ca. 1650 cm^−1^ could imply an interaction between the oxygen atom of the PCL carbonyl and the metal ions [46]. According to the literature [41,47,48,49] and considering that the copper, zinc, and iron elements are in the divalent form, the adsorption mechanism shown in Figure 5 proposes two nitrogen ligands of parallel CS chains and oxygen, the latter either from PCL, or hydroxyl anions at the alkaline media.

The MCP had a Young’s modulus of 212.60 ± 73.69 MPa, a tensile strength of 6.75 ± 0.69 MPa, and a maximum elongation at break of 101.95 ± 0.82%. The tensile strength for the extruded blends of CS with polyester presented relatively low values. A plausible explanation is the immiscibility and inherent incompatibility between CS and polyester, as reported by Correlo et al. [36] with chitosan and polyester blends.

The swelling of the MCP reached equilibrium after 24 h with 85.77%, and afterward, the amount of retained water remained constant (*p* > 0.05) (Appendix E). In a previous report by Wu [22] and Bikiaris et al. [50], high contents of CS in melted blends with polyesters tended to increase water absorption. This was primarily due to the presence of amine and hydroxyl moieties in the CS and their bonding to the water molecules. Nonetheless, a good adsorption capacity could be related to a higher number of available free amine groups, which could also be related to an increased metal ion adsorption capacity [31]. Finally, it is worth mentioning that the erosion of MCP did not change significantly (*p* > 0.05) after 120 h, even though a pH of 4 favors CS dissolution (Appendix E).

### 3.2. Adsorption Kinetics and Equilibrium

**Figure 6:**
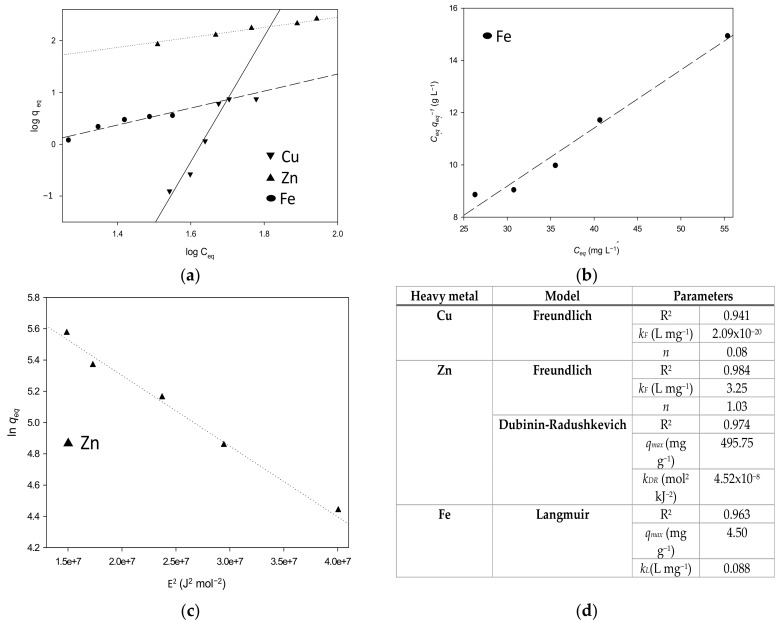
Adsorption experimental data fitted to isotherm models Freundlich (**a**), Langmuir (**b**), Dubinin-Radushkevich (**c**) and their equilibrium parameters (**d**).

The empirical (Freundlich), chemical (Langmuir), and Polanyi’s potential theory-based models (Dubinin-Radushkevich) were fitted to the adsorption data (Figure 6). These models are extensively used to describe sorption reactions of solutes [28]. The equation of Freundlich had a better fit for Cu and Zn (Figure 6), revealing that the adsorbent surface is heterogeneous in a multi-layer adsorption mechanism [1,51]. While the adsorption data for Fe are well-fitted by the Langmuir model, this implies that chemisorption occurred in a monolayer filling the outer interface of MCP (Figure 6). Previous studies conducted with chitosan and modified chitosan-based materials as heavy metal adsorbents also reported that data fitted to both Langmuir and Freundlich isotherms indicate that mono- and multi-layer adsorptions may have occurred [30]. There is also a multi-layer physisorption mechanism for Zn into MCP, supported by the high R^2^ of the fitting to the Dubinin-Radushkevich model (Figure 6), describing pore filling [52]. The initial and final pH remained without significant changes for Zn, while Cu became slightly acidic from a pH of 6.15 ± 0.05 to 5.67 ± 0.19. For Fe, the pH changed from 6.03 ± 0.09 to 6.65 ± 0.10, close to pHzpc (Appendix F). At a pH of 6, Al(III) precipitated rather than adsorbed as it is in the predicted species distribution curves (Appendix C). The order of MCP affinity was Zn and Cu based on the estimated *k_F_*, while comparing *q_max,_* the adsorption of MCP was higher for Zn than Fe (Figure 6).

**Figure 7:**
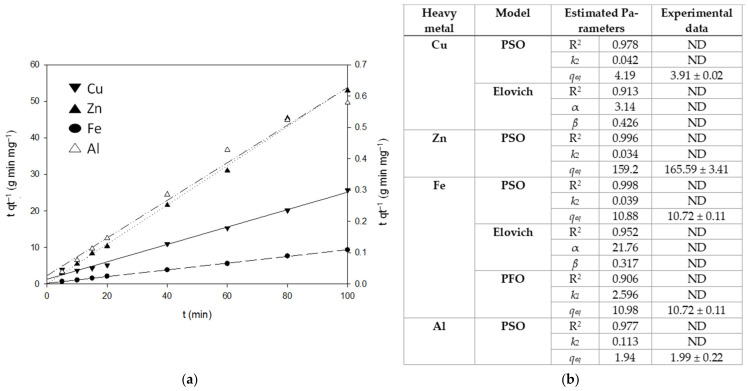
Adsorption experimental data fitted to PSO (**a**) and kinetic parameters for the adsorption of heavy metals in synthetic water into MCP (**b**).

Figure 7 shows the adsorption kinetics for Zn, Fe, Cu, and Al into MCP. Only, in the beginning, MCP adsorbed Pb(II) and Cd(II), which might be due to the competition between metal ions for active sites in CS [1]. After 60 min of treatment, MCP reached the maximum adsorption capacity with almost all the metal ions in the sample. The PSO model fitted better the adsorption data of Cu(II), Zn(II), Fe(II), and Al(III) than Elovich or PFO (R^2^ ≤ 0.9) (Figure 7). The limiting step might be the chemisorption at a certain extent with complexation to the surface, which involves valency forces through the sharing or the exchange of electrons between the adsorbent and adsorbate in an ion-exchange adsorbent/adsorbate mechanism [51,53]. The PSO model estimated *q_eq_* values which are close to the *q_eq_* values determined experimentally (Figure 7). It is worth mentioning that the estimation of the *q_eq_* for each metal ion in a complex mixture solution showed the competition for the functional groups in MCP. The highest *q_eq_* was for Zn (159.25 mg g^−1^) and was 38-fold, 14.7-fold, and 82-fold higher than for Cu, Fe, and Al, respectively (Figure 7). Chen et al. [54] reported a decrease in the adsorption capacity of Zn in the presence of other metal ions in a eucalyptus leaf-based magnetic biosorbent. Herein, the Zn adsorption capacity into MCP was higher than those obtained by other CS-based materials previously reported (Table 1). The affinity and compatibility of CS and PCL with Zn(II) either or both adsorbent or doped materials are well known [21,46,55,56,57,58]. Another plausible explanation for the high adsorption capacity of Zn(II) into MCP is the ion size. Zn has a small size and can diffuse across the inner MCP layers and pores faster than Cd and Pb with larger sizes, occupying the available groups (Section 3.1).

### 3.3. Desorption and Reusability

**Figure 8:**
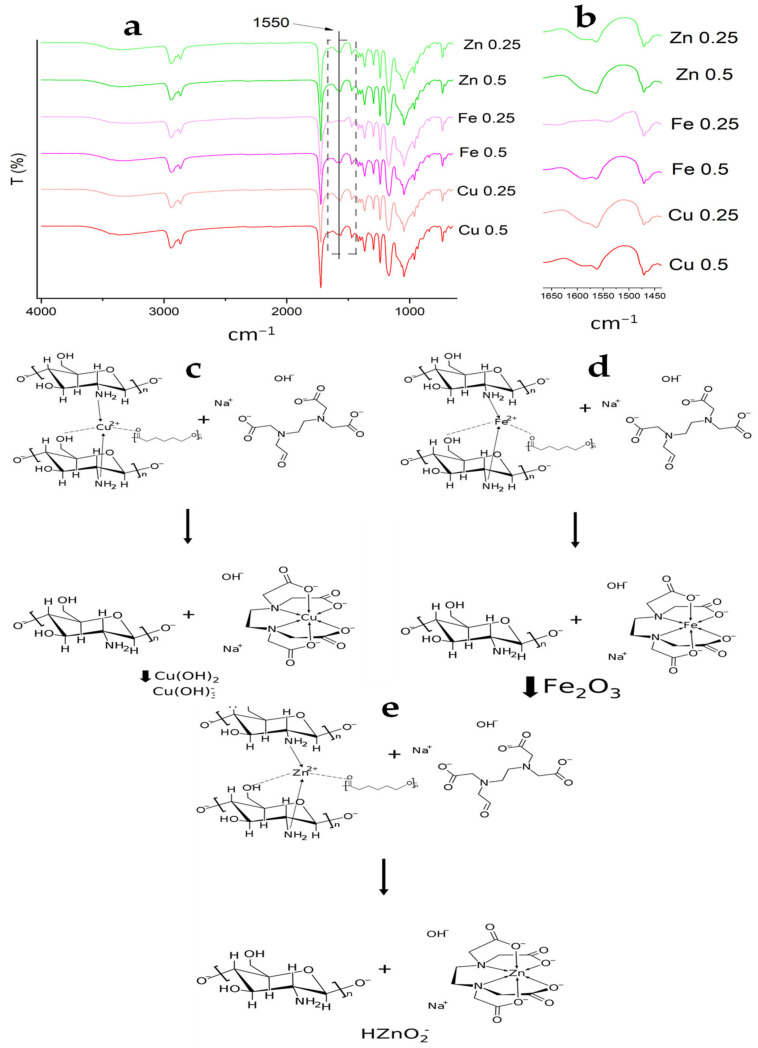
FTIR spectra of MCPA after desorption treatment with an EDTA solution and 0.25 or 0.5 N of NaOH (**a**); 1650–1400 cm^−1^ region (**b**); proposed desorption mechanism for Cu (II) (**c**); Fe (II) (**d**) and Zn (II) (**e**).

The addition of an alkaline EDTA solution favors the formation of highly stable, soluble complexes, thereby desorbing Cu and Fe from MCP. The results suggest that some metal ions precipitated and others might have been chelated by the EDTA molecules (Figure 8) [61]. For zinc ions, anionic species such as HZnO_2_^−^ are formed that also could be chelated by EDTA [41,47,48,49]. On the other hand, the complete desorption of copper might be ascribed to the formation of the bridge complex with CS, which resulted in less stability and easier dissociation [44]. However, the Zn and Fe ions were not completely desorbed after 120 min with EDTA/NaOH 0.5 N and corroborated with the Zn and Fe concentrations in the MCP, which were 46.93 ± 0.08 mg kg^−1^ and 0.50 ± 0.04 mg kg^−1^, respectively. According to Krężel & Maret [48] and Pearson [62], based on the classification of Lewis acids and bases, Zn(II) and Fe(II) are borderline acids with a tendency to form stable complexes with moderately polarizable ligands such as the nitrogen donors from CS. It could explain the difficulty of complete desorption. The proposed adsorption mechanism among CS, Fe, Zn was the formation of complex coordination sites with up to six or seven ligands [45].

**Figure 9:**
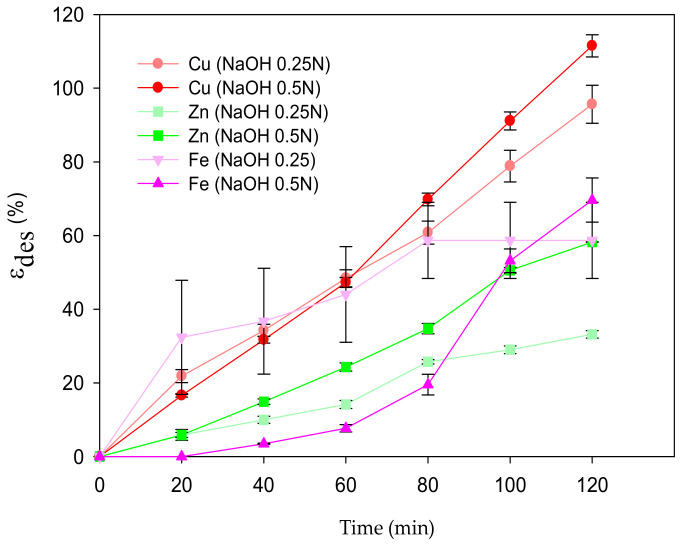
Desorption efficiencies of metal ions from MCPA.

From the ε_des_ shown in Figure 9, after 120 min of treatment, almost 100% of the Cu(II) desorbed with both EDTA solutions (Section 2.5). The 0.5 N concentration of NaOH allowed for a more efficient desorption process (*p* < 0.05) than that with 0.25 N, which contrasts with Wang et al. [27], who proved that a low concentration of NaOH was desirable for full desorption. The *ε_des_* with HCl (0.5 N) reached the highest values of 53.43 ± 9.72%, 11.96 ± 1.94%, and 41.81 ± 12.05% for Fe, Zn, and Al, respectively. Nonetheless, the *E%* after the desorption process in acidic conditions (HCl 0.5 N) was also the highest, representing a disadvantage for the MCP reusability. In the case of the desorption process with HNO_3_, 0.05 N and 0.5 N achieved the complete desorption of Fe without MCP mass loss. Nonetheless, MCP lost its original form and destroyed the pellet, confirming that the adsorption mechanism was mainly due to electrostatic interactions [1]. The highest ε_des_ for Zn was 8.6 ± 1.53% and for Al was 3.23 ± 0.2% with 0.1 N. The ε_des_ values with acidic solutions are shown in Appendix G.

The contaminated MCP with adsorbed heavy metal ions was exposed to a fresh EDTA/NaOH 0.5 N solution and left in agitation for another 2 h to achieve a full desorption of Zn(II). After four changes of the desorption solution, the *ε_des_* achieved was 79.19 ± 0.21% for Zn(II) from the contaminated MCP (Appendix H). The gradient formation resulted in a higher desorption efficiency with the addition of a fresh desorption solution.

**Figure 10:**
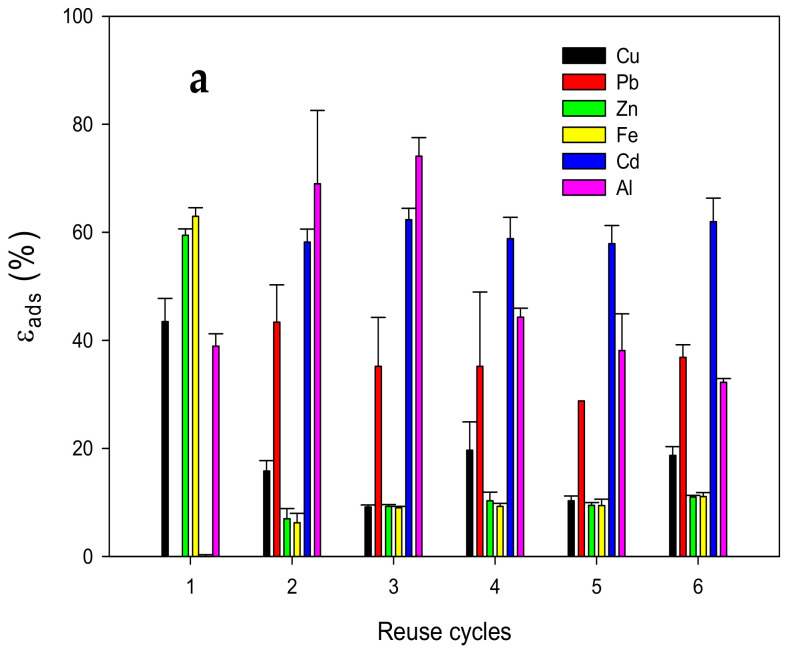

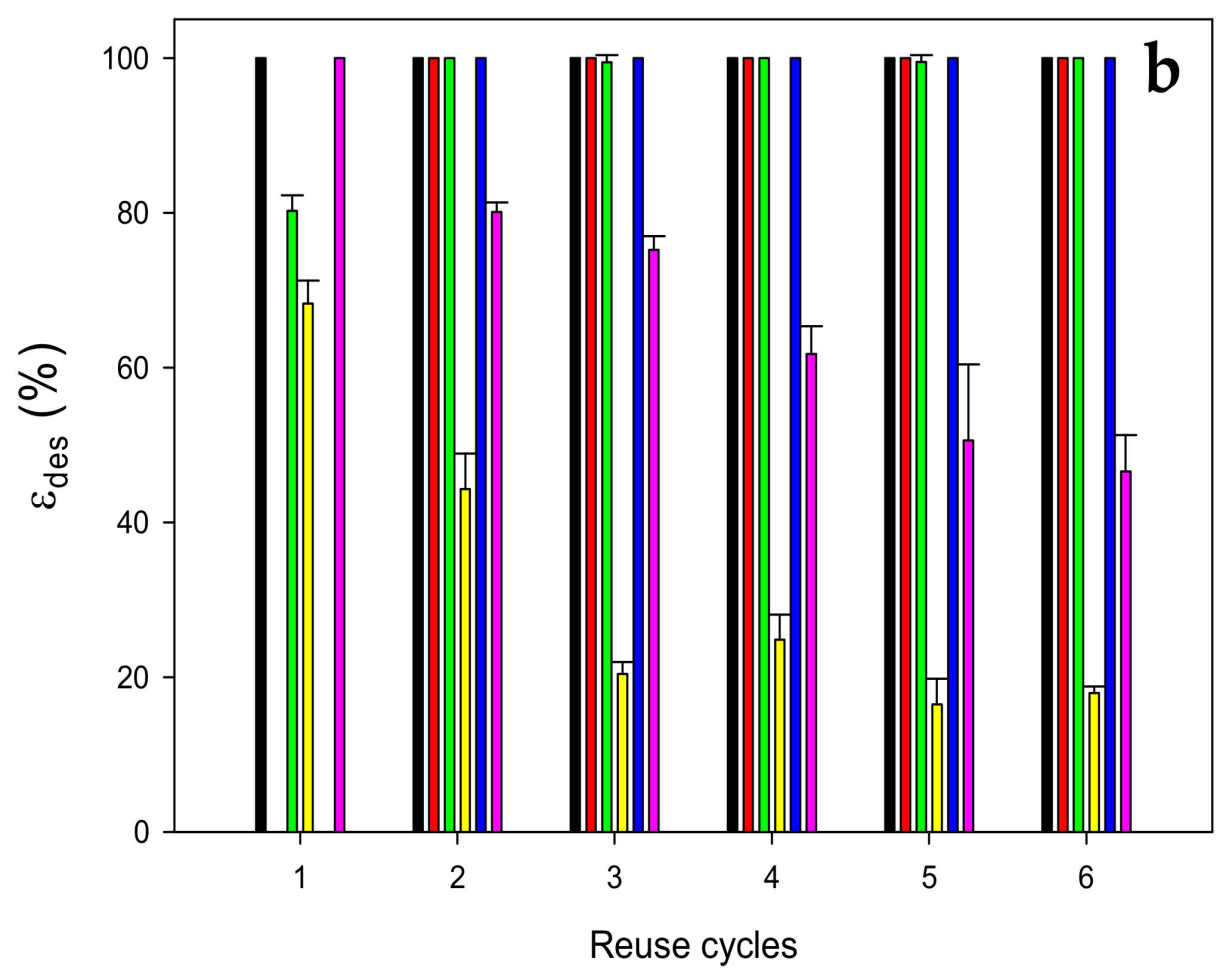
(**a**) Adsorption (*ε_ads_*) and (**b**) desorption (***ε_des_***) efficiencies of Cu (**black**); Pb (**red**); Zn (**green**); Fe (**yellow**); Cd (**blue**) and Al (**pink**).

The reusability of MCP was proven by adding MCP in synthetic solution for an adsorption cycle, and subsequently, desorption was carried out with an EDTA/NaOH 0.5 solution for a total of six cycles (Figure 10). High εads values were observed in the first cycle for Zn(II), Fe(II), Al(III), and Cu(II), which agree with the kinetic studies previously discussed (59.4 ± 1.2%, 62.9 ± 1.5%, 43.4 ± 4.3%, and 38.9 ± 1.2% respectively). The first desorption cycle achieved the release of 100% of the Cu(II) and Al(III) adsorbed in the MCP. Zn(II) and Fe(II) only desorbed 80% and 68%, respectively. In further reuse cycles, the ε_ads_ values of Zn(II), Fe(II), and Cu(II) decreased considerably possibly due to the incomplete desorption of MCP, owing to MCP’s high affinity for these ions. Surprisingly, from the second cycle, the ε_ads_ values of Pb(II) and Cd(II) rose 43.3 ± 6.8% and 62.3 ± 2.1%, respectively, and remained constant until completion of the six cycles. For CS-metal ion complexes studied for the adsorption of other metal ions [63], it was reported that the adsorption efficiency improved because the CS-metal complex acted as a hard base with an affinity for hard acids such as Pb(II) and Cd(II), which explains the adsorption of these ions by Zn(II) and Fe(II) adsorbed in MCP after the first desorption cycle.

### 3.4. Elemental Analysis by EDS

SE micrographs using backscattered electrons signed along with their X-ray diffraction patterns for MCP samples before and after the adsorption treatment showed the main elemental composition of MCP (Appendix I). CS, PCL, and MCP, before the adsorption, were mainly composed of carbon and oxygen. After the adsorption, MCP’s composition changed, and Al and Fe were also detected in the CS fraction, which corroborated the adsorption role of CS in the MCP.

## 4. Conclusions

The understanding of the adsorption mechanism of a mixture of metal ions in water at a pH near the point of zero charge (pHzpc) of the adsorbent composite for reducing electrostatic interactions and metal competition for active groups for chelation led to the improvement of the adsorption process. MCP presents a high selectivity for Zn (165.59 ± 3.41 mg g^−1^) in comparison with other CS-based materials. In addition, the material is capable of removing metal ions from water. Adsorbed heavy metals were successfully desorbed, and six cycles of adsorption-desorption were achieved. Desorbed MCP remains effective for adsorbing metal ions and improves its performance since Pb and Cd were retained after the first adsorption-desorption cycle. Therefore, this composite was able to remove several metal ions in a continuous system and desorb them. This experimental evidence leads to the conclusion of the successful utilization of MCP in several effluent cycles for the removal of metal contaminants. The research in this field finds common grounds in the design of CS-derived and reusable adsorbent materials as an economically attractive, environmentally friendly, and profitable path.

## Figures and Tables

**Table 1:** Comparison of the Zn adsorption capacity into MCP with other CS based materials.

CS Based Composite	*q_e_* (mg g^−1^)/Model	*q_max_* (mg g^−1^)/Model
CS-PCL composite (this work)	Synthetic solution 165.59 ± 3.4 Pseudo-second order	Zn solution 495.75 Dubinin-Radushkevich
CS and CS grafted materials [4]	Zn Solution 151.7 Pseudo-second order	Binary solution of Zn and cationic dye 168.8 with neat CS 290 with succinyl-grafted Tóth
PVA/CS beads [8]	Quaternary solution of Zn, Cu, Pb and Cd 35.71 Pseudo-second order	Zn Solution 83.33 Langmuir
CS coated diatomaceous earth [19]	Zn Solution 130.7 Pseudo-second order	Zn Solution 140.84 Langmuir
CS/poly(ethylene oxide) membrane [59]	Zn Solution 84.43 Pseudo-second order	Zn Solution 117 Langmuir
Activated coconut shell carbon modified with CS [60]	ND	Zn Solution 60.41 Langmuir

ND: Non determined.

**Figure A1:**
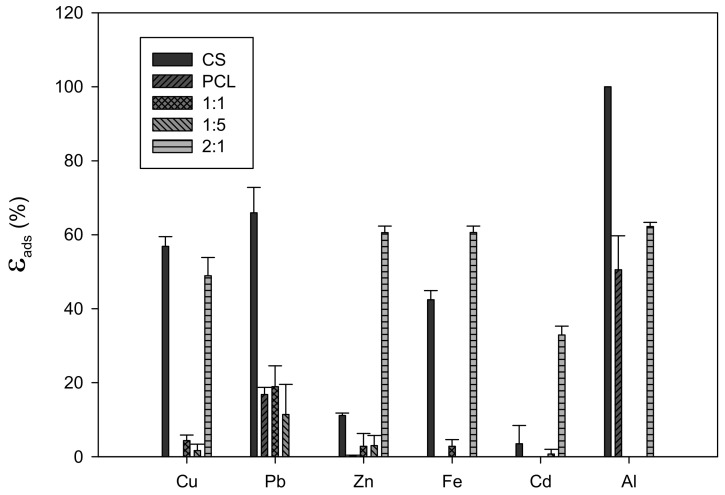
Heavy metal adsorption efficiency of the MCP formulations.

**Figure A2:**
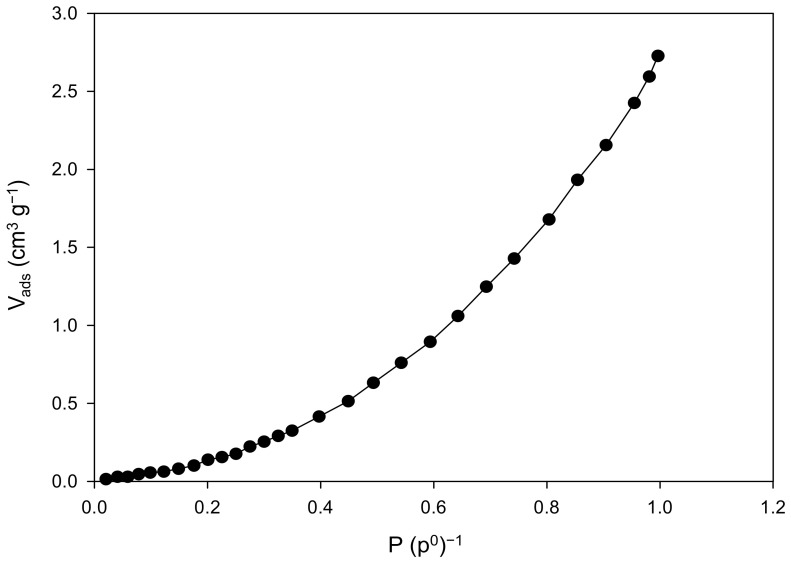
N_2_ physisorption isotherm of MCP.

**Figure A3:**
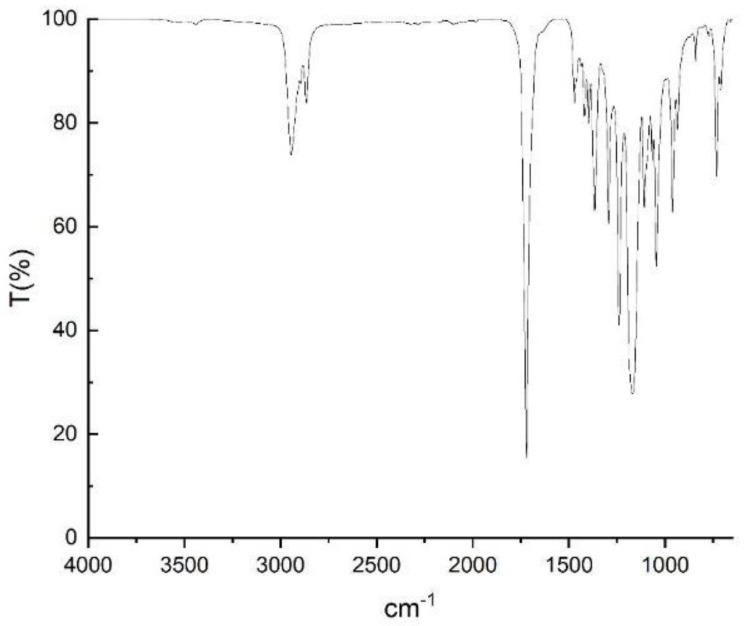
FTIR spectrum of PCL.

**Figure A4:**
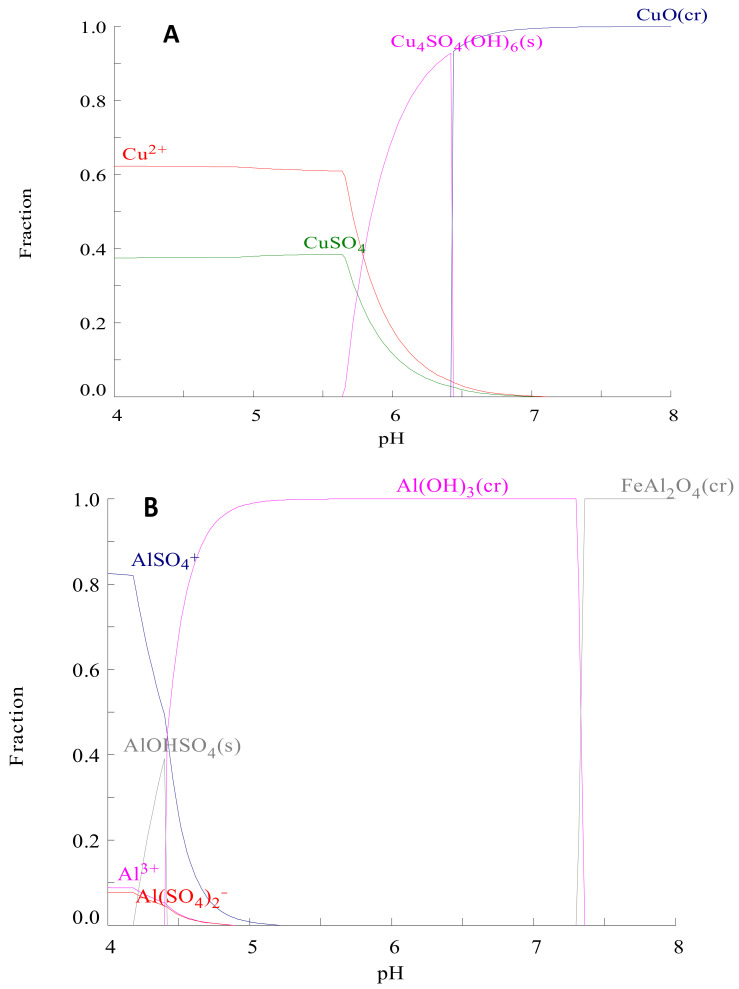
Predicted species distribution curves for (**A**) Cu(II) and (**B**) Al(III) in the synthetic solution as a function of pH at 25 °C, elaborated with the MEDUSA software suite. Concentrations: [Cu(NO_3_)_2_] = 1.6 × 10^−4^ M, [FeSO_4_] = 3.9 × 10^−4^ M, [ZnSO_4_] = 5.1 × 10^−3^ M, [Cd(NO_3_)_2_] = 8.8 × 10^−6^ M, [Pb(NO_3_)_2_] = 2.4 × 10^−5^ M and [Al(NO_3_)_3_] = 1.5 × 10^−4^ M.

**Figure A5:**
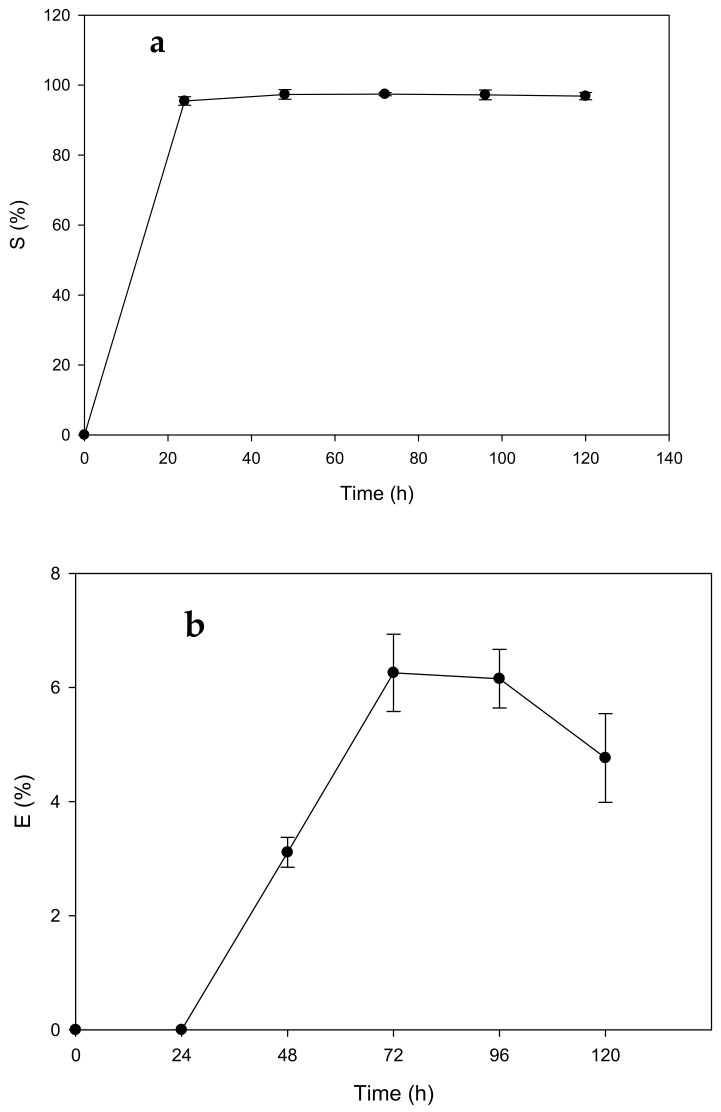
Time course of water swelling at 25 °C and RH of 45% (**a**) and MCP erosion behavior in acidic solution (pH 4) (**b**).

**Figure A6:**
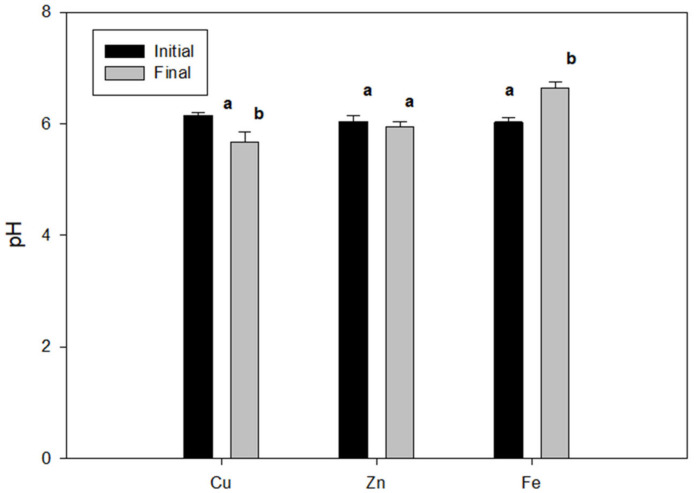
The pH of adsorption equilibrium solutions. Initial and final histograms with different letter mean significant differences according to Tukey-Kramer multiple comparison test (*p* ≤ 0.05).

**Figure A7:**
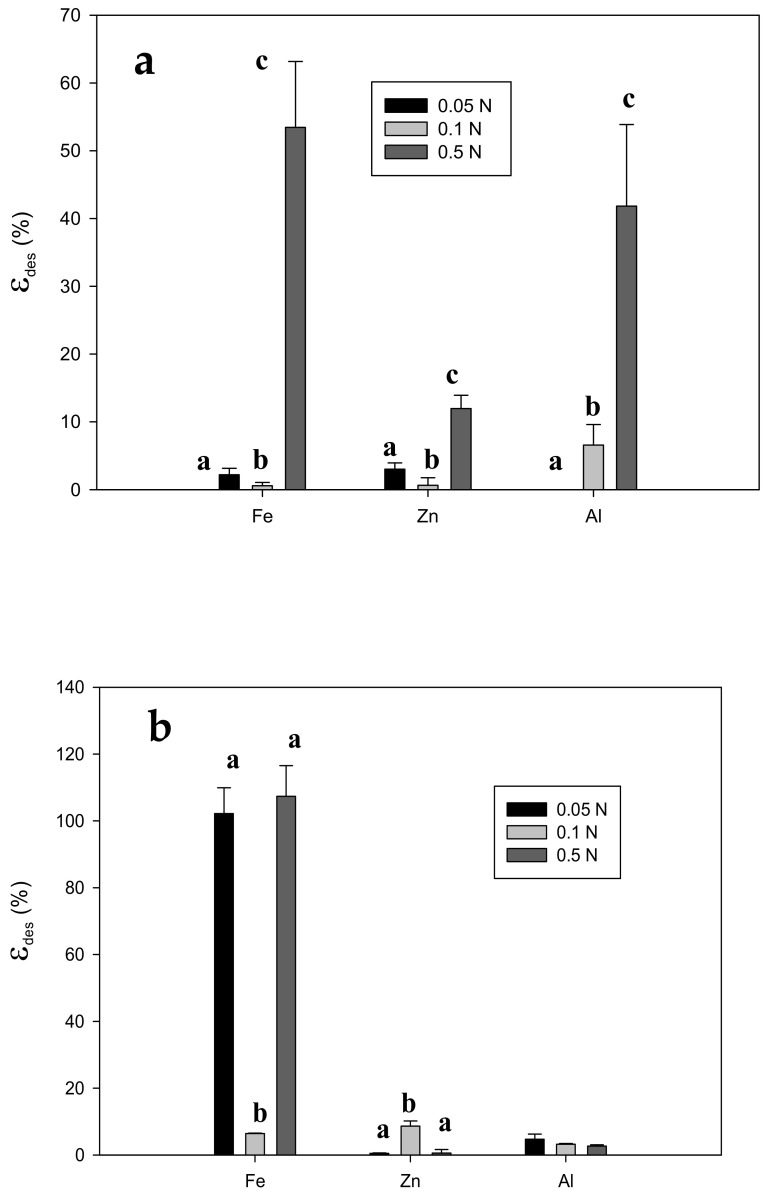

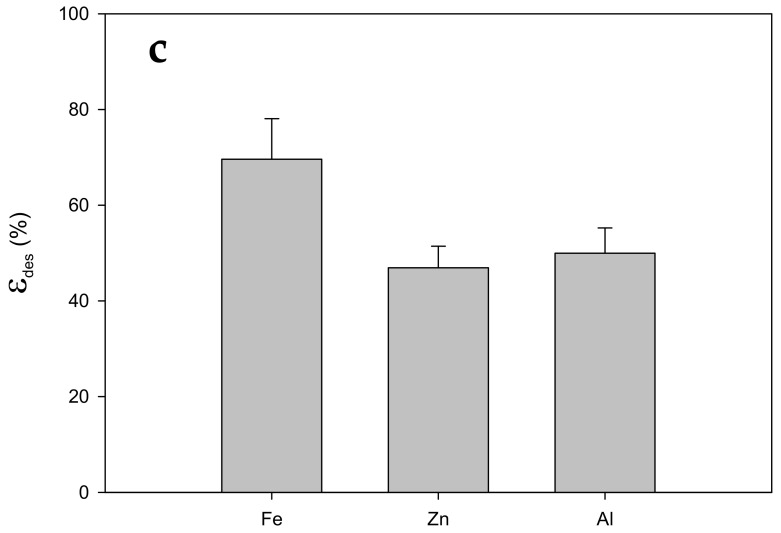
The εdes of Fe, Zn and Al with 10 mL of HCl (**a**), HNO_3_ (**b**) and EDTA 15% + NaOH 0.5 N (**c**) solutions after 120 min at agitation of 50 rpm. Different letters means differences according to Tukey-Kramer multiple comparison test (*p* < 0.05).

**Figure A8:**
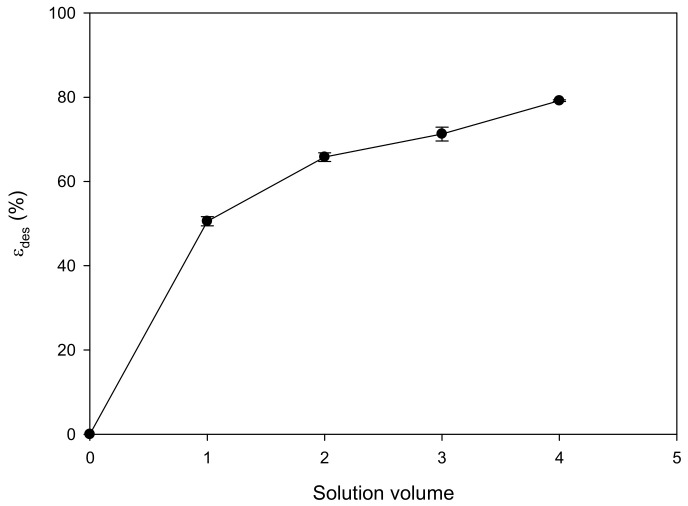
The desorption efficiency of Zn(II) ions changing the desorption solution for fresh EDTA/NaOH 0.5 N volume.

**Figure A9:**
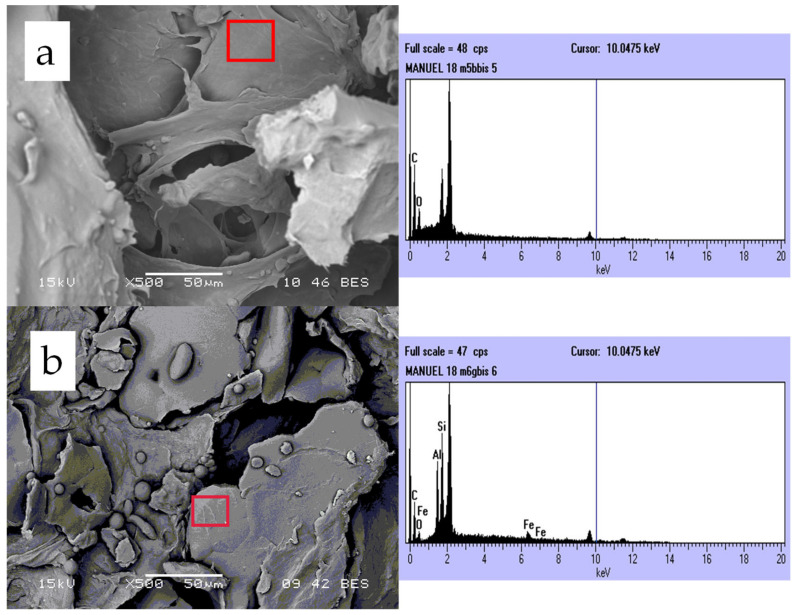
EDS for MCP sample before (**a**) and after (**b**) water treatment.

## Data Availability

The data presented in this study are available on request from the corresponding author.

## Appendix A

Appendix A shows the adsorption efficiency of the MCP formulations, CS and PCL for 2 h at an agitation of 5 rpm with 10 mL of synthetic solution (Figure A1).

## Appendix B

Appendix B shows the N_2_ physisorption isotherms of MCP (Figure A2).

## Appendix C

Appendix C shows the FTIR spectrum of PCL (Figure A3).

## Appendix D

Appendix D (Figure A4) shows the predicted species distribution curves for (A) Cu(II) and (B) Al(III) in synthetic solution as a function of pH at 25 °C, elaborated with the MEDUSA software suite. Concentrations: [Cu(NO_3_)_2_] = 1.6 × 10^−4^ M, [FeSO_4_] = 3.9 × 10^−4^ M, [ZnSO_4_] = 5.1 × 10^−3^ M, [Cd(NO_3_)_2_] = 8.8 × 10^−6^ M, [Pb(NO_3_)_2_] = 2.4 × 10^−5^ M and [Al(NO_3_)_3_] = 1.5 × 10^−4^ M.

## Appendix E

Appendix E (Figure A5) shows the time course of water swelling at 25 °C and an RH of 45% (a) and MCP erosion behavior in an acidic solution (pH 4) (b).

## Appendix F

Appendix F (Figure A6) shows the pH of adsorption equilibrium solutions. Histograms with different letters among metal ions mean there are significant differences according to the Tukey-Kramer multiple comparison test (*p* ≤ 0.05).

## Appendix G

Appendix G (Figure A7) shows the εdes of Fe, Zn, and Al with 10 mL of HCl (a), HNO3 (b), and EDTA 15% + NaOH 0.5 N (c) solutions after 120 min at an agitation of 50 rpm. * Significantly different according to the Tukey-Kramer multiple comparison test (*p* < 0.05).

## Appendix H

Appendix H (Figure A8) shows the desorption efficiency of Zn(II) ions changing the desorption solution for fresh EDTA/NaOH 0.5 N volume.

## Appendix I

Appendix I (Figure A9) shows the EDS for the MCP sample before (a) and after (b) water treatment.
